# Delayed cerebral abscess caused by *Scedosporium apiospermum* following fecal pit near-drowning

**DOI:** 10.1016/j.mmcr.2026.100786

**Published:** 2026-04-18

**Authors:** Han Sheng, Deng Hongfei, Feng Ke, Guo Xiujuan, Xiao Chunxia

**Affiliations:** a Department of Emergency Medicine, Linfen People's Hospital, Linfen, Shanxi, China; b Graduate School of Changzhi Medical College, Changzhi, Shanxi, China; c Clinical Microbiology Laboratory, Linfen People's Hospital, Linfen, Shanxi, China

**Keywords:** Drowning, *Scedosporium apiospermum*, Brain abscess, Pulmonary infection, Voriconazole

## Abstract

*Scedosporium apiospermum* is an opportunistic mold commonly found in sewage and contaminated water and may cause invasive infection after near-drowning, even in immunocompetent individuals. We report a 57-year-old man who developed severe aspiration pneumonia after septic pit near-drowning and initially improved with supportive treatment. On day 49, he presented with headache, and imaging revealed a left frontal brain abscess. Surgical intervention combined with voriconazole therapy resulted in a favorable outcome.

## 1 Introduction

*Scedosporium apiospermum* is a filamentous opportunistic fungus widely distributed in soil, sewage, and contaminated water [1]. It is capable of causing a broad spectrum of clinical manifestations in both immunocompetent and immunocompromised hosts, ranging from asymptomatic colonization to invasive infection [2]. In immunocompetent individuals, infection is usually localized or limited to colonization; however, under high-risk exposure conditions such as near-drowning—particularly following contact with heavily contaminated water—progression to invasive disease may occur [3].Invasive *Scedosporium* infections following near-drowning are often characterized by delayed onset and nonspecific early clinical manifestations, which can lead to misdiagnosis or delayed diagnosis [4]. Although central nervous system involvement is relatively uncommon, once present it typically manifests as brain abscess or disseminated infection and is associated with rapid progression and high mortality [5].Because fecal pit near-drowning represents an extreme form of contaminated water exposure and reports of subsequent invasive fungal infection remain limited, we present a case of delayed-onset *S. apiospermum* brain abscess following fecal pit near-drowning. This report aims to highlight the clinical course, diagnostic challenges, and therapeutic considerations of this rare but life-threatening condition.

## 2 Case presentation

**Fig. 1:**
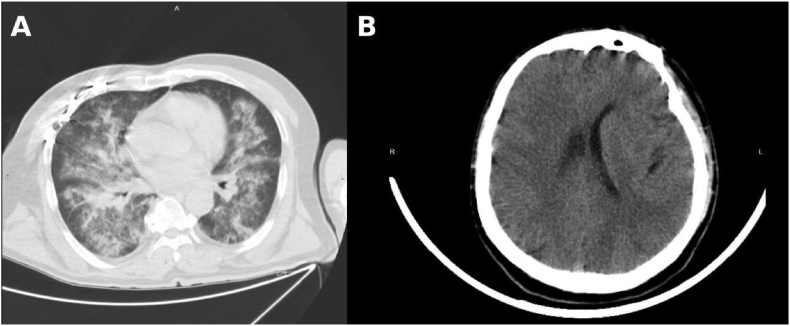
**Initial chest and brain imaging on admission (Day 0).** (A) Chest computed tomography (CT) performed on Day 0 showing diffuse bilateral pulmonary infiltrates consistent with severe aspiration pneumonia following fecal pit near-drowning. (B) Brain CT obtained on admission demonstrating no acute intracranial abnormalities.

A 57-year-old man was included in this case. In this report, the day on which the patient accidently fell into a fecal pit and experienced near-drowning exposure was defined as Day 0 (D0). Upon rescue, he presented with impaired consciousness, which gradually improved; however, sighing respirations persisted. He was initially treated at a local hospital and was subsequently transferred to our institution for further management. After admission, he required endotracheal intubation and mechanical ventilation support.On physical examination, his temperature was 37.1 °C, heart rate 106 beats/min, blood pressure 98/51 mmHg, and respiratory rate 18 breaths/min with sighing respiration. Peripheral cyanosis was noted, and bilateral moist rales were audible on lung auscultation.Chest and cranial CT performed prior to admission revealed periventricular cerebral infarction, sinusitis, bilateral pneumonia, and old fractures of multiple right ribs (Fig. 1).Arterial blood gas analysis showed: pH 7.156, PaO_2_ 37.6 mmHg, PaCO_2_ 46.8 mmHg, K^+^ 3.6 mmol/L, Na^+^ 141 mmol/L, standard base excess (SBE) 14.5 mmol/L, glucose 16.5 mmol/L, lactate 8 mmol/L, and total bilirubin (TBIL) 67 μmol/L.

After admission, the patient was diagnosed with aspiration pneumonia, acute type I respiratory failure, metabolic acidosis, and hyperlactatemia. He also had a history of old cerebral infarction and prior internal fixation for a right rib fracture. Empirical anti-infective therapy was initially administered with intravenous piperacillin (4 g every 12 h). Based on sputum culture and antimicrobial susceptibility testing, the regimen was subsequently adjusted to piperacillin/tazobactam (4.5 g every 12 h), with a total antibacterial treatment duration of 14 days.Concomitant glucocorticoid therapy was given, starting with hydrocortisone 200 mg/day. After 3 days, this was changed to methylprednisolone 80 mg/day and gradually tapered to 40 mg/day, with a total corticosteroid course of 11 days. During comprehensive supportive treatment, the patient developed transient fever. Following treatment adjustments, his respiratory status gradually improved, allowing successful weaning from mechanical ventilation and extubation. His overall condition improved, and he was discharged after staged inpatient treatment. During hospitalization, dynamic changes in arterial blood gas parameters, oxygenation index, complete blood count, C-reactive protein, and sputum culture results were used to guide adjustments in the anti-infective regimen.

**Fig. 2:**
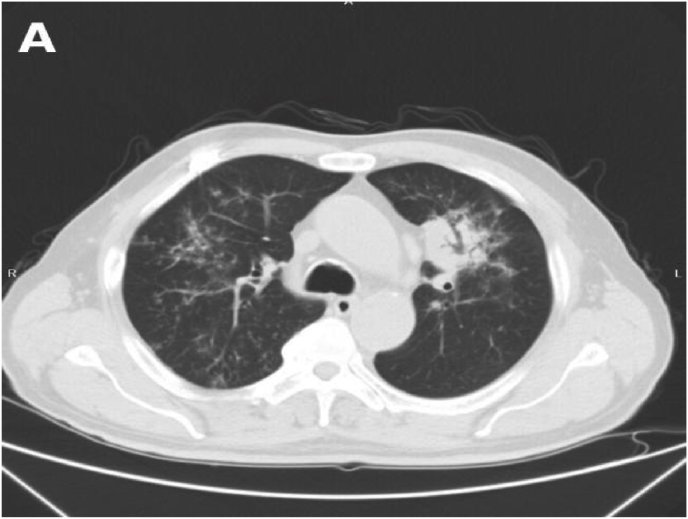
**Follow-up chest CT on Day 26 (D26).** (A) Chest CT obtained on Day 26 (D26) showing progression of the lesion in the left upper lobe, with partial resolution of infiltrates in both lower lobes compared with initial imaging.

On Day 26 after the initial exposure, the patient returned for outpatient follow-up without obvious discomfort. Follow-up chest CT revealed progression of the lesion in the left upper lobe, with partial resolution of infiltrates in both lower lobes (Fig. 2).

**Fig. 3:**
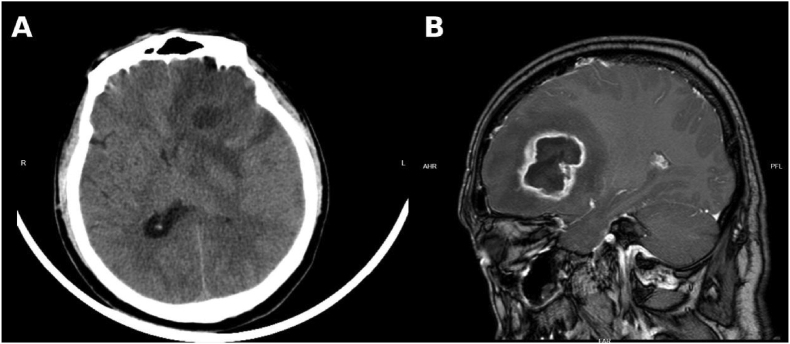
**Neuroimaging findings on Day 49 (D49).** (A) Brain CT obtained on Day 49 (D49) demonstrating a space-occupying lesion in the left frontal lobe with surrounding edema. (B) Contrast-enhanced magnetic resonance imaging (MRI) revealing a ring-enhancing lesion consistent with a brain abscess.

**Fig. 4:**
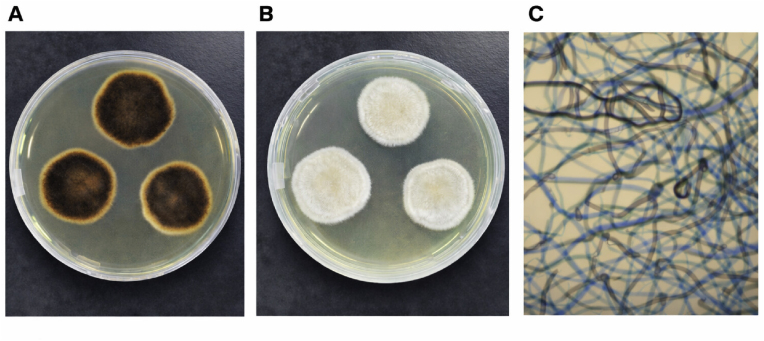
Colony morphology and microscopic characteristics of the isolated strain cultured on Sabouraud dextrose agar (SDA). (A) Reverse colony morphology after 48 h of incubation on SDA, showing dark brown to black pigmentation. (B) Obverse colony morphology after 72 h of incubation on SDA, presenting white, woolly colonies. (C) Microscopic morphology showing hyaline, septate, branching hyphae with conidia arising from the tips of conidiophores (lactophenol cotton blue stain, × 400).

On Day 49, the patient presented again with acute onset of headache and dizziness. Brain CT and magnetic resonance imaging (MRI) demonstrated a space-occupying lesion in the left frontal lobe with surrounding edema, raising suspicion for a brain abscess (Fig. 3). Emergency craniotomy with abscess excision was performed, during which yellow-green purulent material was obtained.Clinical specimens were inoculated onto Sabouraud dextrose agar (SDA) and incubated at an appropriate temperature to observe colony morphology. After 48 h of incubation, the reverse side of the colonies showed dark brown to black pigmentation. After 72 h, the obverse side presented white, woolly colonies. Fresh colonies were subsequently selected and identified using the VITEK MS microbial identification system (bioMérieux, France) in the microbiology laboratory of Linfen People's Hospital. The isolate was identified as *Scedosporium apiospermum***(**Fig. 4**)**.

**Fig. 5:**
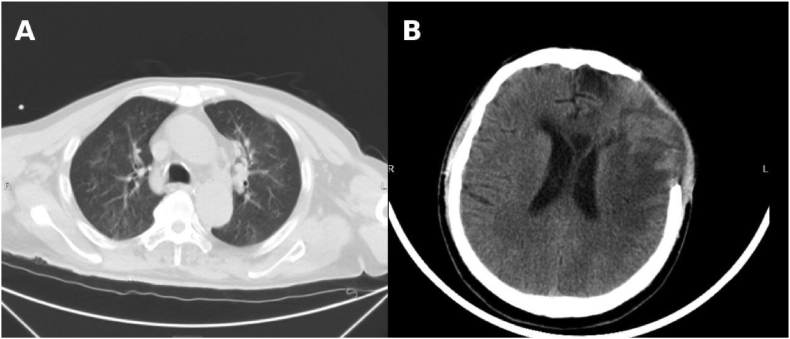
**Follow-up imaging after surgical intervention and voriconazole therapy.** (A) Follow-up chest CT after voriconazole therapy showing marked improvement of pulmonary lesions. (B) Postoperative brain CT demonstrating stable postoperative changes without evidence of new intracranial lesions.

Following microbiological confirmation, antifungal therapy with voriconazole was initiated via a nasogastric tube, consisting of a loading dose of 400 mg every 12 h for the first 24 h, followed by a maintenance dose of 200 mg every 12 h for 14 days. As the abscess had been surgically excised and no evidence of disseminated infection was identified, a short-course antifungal regimen was considered appropriate. The patient remained clinically stable with radiological improvement.Postoperatively, the patient developed surgical site hemorrhage with intraventricular extension and underwent a second operation for hematoma evacuation. With comprehensive treatment, his condition gradually improved, and he was discharged in stable condition. Follow-up imaging after antifungal therapy demonstrated marked improvement of pulmonary lesions and stable postoperative intracranial findings without evidence of new lesions (Fig. 5).

At discharge, diagnoses included left frontal lobe brain abscess, postoperative intracranial hematoma, diffuse cerebral edema, bilateral pneumonia, and recovery phase following toxic gas exposure. No clinical or radiological evidence of recurrence was observed at 1-month follow-up.

## 3 Discussion

This case indicates that exposure to highly contaminated water, such as a fecal pit, carries a risk of delayed invasive *Scedosporium apiospermum* infection even in immunocompetent individuals [6]. Unlike typical drowning events, sewage- or feces-contaminated water contains a high burden of environmental fungi, which may facilitate subsequent colonization and progression to invasive disease [7].In the present case, although the patient was asymptomatic at follow-up on Day 26, chest imaging already demonstrated progression of pulmonary lesions. Based on previous reports, this stage may represent pulmonary colonization or early invasive infection. Pulmonary colonization has been described as fungal balls within preexisting cavities or dilated bronchi, whereas invasive disease may present with halo signs, crescent-shaped cavities, focal consolidation, or necrotizing pneumonia [8,9]. Owing to the lack of specific early clinical manifestations, recognition of invasive infection at this stage is often difficult and may be delayed [10].

Pulmonary involvement is the most common manifestation of *Scedosporium apiospermum* infection, and dissemination to joints (most frequently the knee) and the eyes has also been reported [11]. Central nervous system involvement is relatively uncommon; however, once it occurs, it most often presents as brain abscess and is associated with rapid disease progression and poor prognosis [12]. In the present case, the patient developed neurological symptoms on Day 49 and was diagnosed with a brain abscess, suggesting a progression from pulmonary colonization or localized infection to invasive central nervous system disease, a pattern that is consistent with previous reports [11]. Notably, *Scedosporium apiospermum* is widely distributed in soil and contaminated water, and even in immunocompetent hosts, near-drowning or exposure to heavily polluted water may serve as an important trigger for invasive infection [13].

From a diagnostic perspective, *Scedosporium apiospermum* infection lacks specific clinical manifestations, and results from conventional culture and histopathological examination are often obtained late, which may delay timely treatment [12]. In recent years, studies have suggested that detection of β-D-glucan in serum or cerebrospinal fluid may serve as an adjunctive diagnostic tool in selected cases, with positive results sometimes appearing earlier than those obtained by traditional methods; however, interpretation still requires integration with clinical and imaging findings [10]. In the present case, the final diagnosis relied on culture of surgical specimens, highlighting the importance of actively obtaining etiological evidence in patients with suspected central nervous system involvement. During the initial hospitalization, the patient primarily presented with severe aspiration pneumonia and acute respiratory failure, without clear evidence suggestive of invasive fungal infection; therefore, empirical antifungal therapy was not initiated.

Currently, multiple studies and consensus statements consistently support voriconazole as the first-line treatment for *Scedosporium apiospermum* infection, as its in vitro antifungal activity is superior to that of amphotericin B and it demonstrates good penetration across the blood–brain barrier [14,15]. In contrast, amphotericin B often exhibits intrinsic resistance against this organism, resulting in limited clinical efficacy [15]. In this case, following definitive etiological diagnosis, timely combination of surgical resection and voriconazole antifungal therapy led to a favorable outcome, further supporting the effectiveness of this treatment strategy in cases with central nervous system involvement.

In addition, this case underscores the importance of heightened awareness regarding the timing of diagnosis and treatment of invasive fungal infections in near-drowning patients. Previous studies have shown that after exposure to highly contaminated water, invasive fungal infection may occur even in immunocompetent hosts, with an incubation period typically ranging from 7 to 10 days, followed by rapid disease progression [6,7]. Because early clinical manifestations are nonspecific, infection may be overlooked due to transient improvement in imaging or laboratory findings, leading to delayed diagnosis and treatment.

Therefore, in patients with a clear history of exposure to contaminated water, close radiological follow-up is warranted even when initial clinical stabilization is observed. When indicated, early fungal culture, β-D-glucan testing, and molecular diagnostic evaluations should be performed to assess the etiology [10]. Once invasive fungal infection is strongly suspected, prompt initiation of targeted antifungal therapy is essential to reduce disease progression and the risk of central nervous system dissemination, thereby improving patient prognosis [6].

## 4 Conclusion

Exposure to heavily contaminated water, such as fecal pit near-drowning, may result in delayed invasive *Scedosporium apiospermum* infection with potential CNS involvement. These infections often present insidiously but can progress rapidly, necessitating heightened clinical vigilance. Early recognition, timely pathogen identification, and combined surgical intervention with appropriate antifungal therapy are critical for improving patient prognosis.

## CRediT authorship contribution statement

**Han Sheng:** Writing – original draft, Software, Resources. **Deng Hongfei:** Writing – review & editing. **Feng Ke:** Formal analysis, Data curation. **Guo Xiujuan:** Writing – review & editing, Project administration, Data curation, Conceptualization. **Xiao Chunxia:** Data curation, Methodology.

## Ethical Form

Written informed consent was obtained from the patient for publication of this case report and accompanying images. This study was conducted in accordance with the Declaration of Helsinki.

The statements on funding, conflict of interest and consent need to be submitted via our Ethical Form that can be downloaded from the submission site www.ees.elsevier.com/mmcr. **Please note that your manuscript will not be considered for publication until the signed Ethical Form has been received.**

## Ethics statement

This case report was conducted in accordance with the ethical standards of the institutional research committee and with the 1964 Helsinki Declaration and its later amendments or comparable ethical standards.

Written informed consent was obtained from the patient for publication of this case report and accompanying images. A copy of the written consent is available for review by the editorial office of this journal.

## Conflict of interest

The authors declare that there are no conflicts of interest.
